# Tumour-derived exosomes as a signature of pancreatic cancer - liquid biopsies as indicators of tumour progression

**DOI:** 10.18632/oncotarget.13973

**Published:** 2016-12-16

**Authors:** Zarin Nuzhat, Vyjayanthi Kinhal, Shayna Sharma, Gregory E. Rice, Virendra Joshi, Carlos Salomon

**Affiliations:** ^1^ Exosome Biology Laboratory, Centre for Clinical Diagnostics, University of Queensland Centre for Clinical Research, Royal Brisbane and Womens Hospital, The University of Queensland, Brisbane QLD 4029, Australia; ^2^ Department of Obstetrics and Gynecology, Ochsner Baptist Hospital, New Orleans, Louisiana, USA; ^3^ Ochsner Clinic Foundation, New Orleans, Louisiana, USA

**Keywords:** exosomes, pancreatic cancer, biomarkers

## Abstract

Pancreatic cancer is the fourth most common cause of death due to cancer in the world. It is known to have a poor prognosis, mostly because early stages of the disease are generally asymptomatic. Progress in pancreatic cancer research has been slow, leaving several fundamental questions pertaining to diagnosis and treatment unanswered. Recent studies highlight the putative utility of tissue-specific vesicles (i.e. extracellular vesicles) in the diagnosis of disease onset and treatment monitoring in pancreatic cancer. Extracellular vesicles are membrane-limited structures derived from the cell membrane. They contain specific molecules including proteins, mRNA, microRNAs and non-coding RNAs that are secreted in the extracellular space. Extracellular vesicles can be classified according to their size and/or origin into microvesicles (∼150-1000 nm) and exosomes (∼40-120 nm). Microvesicles are released by budding from the plasmatic membrane, whereas exosomes are released via the endocytic pathway by fusion of multivesicular bodies with the plasmatic membrane. This endosomal origin means that exosomes contain an abundance of cell-specific biomolecules which may act as a fingerprint of the cell of origin. In this review, we discuss our current knowledge in the diagnosis and treatment of pancreatic cancer, particularly the potential role of EVs in these facets of disease management. In particular, we suggest that as exosomes contain cellular protein and RNA molecules in a cell type-specific manner, they may provide extensive information about the signature of the tumour and pancreatic cancer progression.

## INTRODUCTION

Pancreatic cancer ranks fourth in causes of deaths due to cancer in the world [1]. According to the American Cancer Society, about 53,000 people will be diagnosed with pancreatic cancer in 2016 in the United States alone. It is also estimated that 41,750 deaths will occur due to pancreatic cancer in the US each year [2]. On average, the management of one pancreatic cancer patient in the U.S. will cost a total of $65,000 [3]. Pancreatic cancer is known to have a poor prognosis, mostly due to the fact that early stages of the cancer are generally asymptomatic. As a consequence diagnosis is usually made by the time it has metastasised, leading to poor patient outcomes with a 5-year survival rate of ∼5 percent for pancreatic ductal adenocarcinoma (PDAC) [4]. Currently, there are no consistently reliable biomarkers or imaging modalities to accurately diagnose, classify, and predict the biological behavior of pancreatic tumours. Although the advancement of traditional imaging methods has improved diagnosis, modalities currently in practice often fail to consistently and accurately predict the metastatic behaviour of the initial lesion in the early stages. Meanwhile, the advent of biomarkers as a diagnostic modality has been promising, but is yet to yield consistent results with high specificity and sensitivity in the clinical setting. Therefore, it is imperative to develop new and improved strategies to detect initial lesions in the early stages of pancreatic cancer with greater diagnostic sensitivity *in vitro*.

Extracellular vesicles (EVs) may serve as a unique strategy for monitoring and managing disease status. The past decade has observed an extraordinary explosion of research in the field of EVs, particularly with regards to exosomes. Exosomes are very stable membrane vesicles that are released from a wide range of cells, including cancer cells. They are distinguished by their size (∼40-120 *nm*). and endosomal origin. Tumour exosomes play a role in cell-to-cell communication between the tumour and contiguous organs. They may also be involved in more distal interactions which include the trafficking of tumour-derived exosomes into biological fluids and subsequently into remote sites of metastasis to prepare a pre-metastatic niche. As the content of exosomes is cell type specific, we suggest that exosomes may provide a unique ‘signature’ of metastatic progression as well as the metabolic status of the tumour. This signature may be invaluable in not only detecting pancreatic cancer in the early stages, but also in developing a prognosis for potential metastases. This may hence aid the development of more effective management plans. Given their stability and abundance in a wide range of biological fluids [5], exosomes are a viable candidate to act as a non-invasive biopsy of the tumour mass. Thus, the aim of this brief commentary is to review the current body of knowledge pertaining to the diagnosis and treatment of pancreatic cancer, particularly with regards to exosomes. Furthermore, this review will discuss the potential role of exosomes in pancreatic cancer progression.

## CURRENT SHORTCOMINGS IN THE DIAGNOSIS AND TREATMENT OF PANCREATIC CANCER

Diagnosis of pancreatic cancer currently centers around imaging with emerging use of biomarkers. However, classic imaging methods are proving increasingly ineffective for the early diagnosis of malignant tumours. The trans-abdominal ultrasound offers little diagnostic benefit particularly for small lesions in the early stage [6]. Endoscopic ultrasounds (EUS), a classic diagnostic method, have a tendency to yield false positive diagnoses [7]. The endoscopic ultrasound with fine needle aspiration (EUS-FNA) provides greater sensitivity and specificity, although accuracy is weakened when the patient also has chronic pancreatitis. In such cases, EUS-FNA provides little benefit for determining malignancy [8]. Furthermore, computed tomography (CT) scans often cannot distinguish alterations in pancreatic morphology and structure, thus are also inadequate for detecting small lesions [9].

However, new imaging modalities such as helical CT scans offer some improvements, including greater contrast and distinguishing abilities [9]. Molecular tracers such as F-fluorodeoxyglucose used in positron emission tomography (PET) and CT imaging hold promise as a powerful diagnostic tool for potentially malignant tumours, although a high false-positive rate in hyperglycaemic patients must be addressed [10].

Biomarkers may be a more sensitive tool for detecting pancreatic cancer in the early stages. For example, carcinoembryonic antigen (CEA) and cancer antigen 19-9 (CA19-9) could potentially diagnose malignant tumours with some accuracy. These are currently used in practice alongside imaging. However, sensitivity for malignancy must be improved, particularly for CA19-9 [11]. The use of CA19-9 as a biomarker is limited by the presence of significantly high levels of CA19-9 in benign pancreatic diseases and normal CA19-9 levels in patients with pancreatic tumours in the early stages [12]. Meanwhile, a meta-analysis of CEA as a diagnostic tool found that for PDAC, the median sensitivity was 54% while the median specificity was 79%. Furthermore, CEA lacked specificity for PDAC as it was also overexpressed in other tumours, such as colorectal tumours [13].

Upon diagnosis by modalities currently in practice, the prognosis for the patient is generally poor. This is because current methods are inaccurate and non-specific for early lesions. Thus by the time of diagnosis, the lesion has metastasised to a significant extent. Hence current treatment and management methods focus on prolonging survival. However, complete elimination of the metastatic capacity of the cancer is often not possible and survival rates are poor. Although surgical resection may heighten quality of life for the patient, a significant survival advantage has only been observed in a minority of pancreatic cancer patients [14]. The survival advantage provided by surgery can be improved greatly by the use of adjuvant therapy. Emerging adjuvant chemotherapy plans may improve overall quality of life and prolong survival by several months, and in some cases, years. However, adjuvant therapies may only be effective for a subset of patients with particular aetiologies [14].

Thus, due to the weakness of current diagnostic modalities, pancreatic cancer patients may only experience limited benefits from emerging and improved surgical and treatment plans. It is hence imperative that diagnostic methods which can offer high specificity, reliability and sensitivity for early lesions are developed. Recent studies highlight the putative utility of tissue-specific nanovesicles (*e.g.* exosomes) in the diagnosis of disease onset and treatment monitoring

## EXOSOMES: A SPECIFIC TYPE OF EXTRACELLULAR VESICLES

Understanding the distinct role of exosomes in intercellular signaling has been a significant step in further elucidating mechanisms of cell-to-cell communication. Exosomes are classified as EVs, but can be distinguished from other EVs through a number of characteristics [15]. For one, exosomes have a distinctive size range of ∼40 - 120 *nm*, where microvesicles (MVs) and apoptotic bodies can vary between 50-1000 *nm* and 800-5000 *nm* respectively. Apoptotic bodies are derived from the cell as fragments of dying cells while MVs bud from the plasma membrane [16]. However, exosomes are notable in their biogenesis because they are derived from the endocytic pathway following the inward budding of MVs. MVs fuse with the plasma membrane and thereby release exosomes into the extracellular environment through exocytosis [15]. This distinctive subcellular origin means exosomes are enriched with a notable array of proteins, including TSG101, CD63, CD81, and CD9. This range of protein content is markedly more diverse than that of apoptotic bodies [5]. Although the mechanism of packaging is yet to be fully understood, the content of exosomes includes a diverse range of signaling molecules including cell adhesion molecules, growth factor receptors, and heat shock proteins. Following exocytotic release, the cargo of exosomes becomes important in shaping the activity of neighbouring cells or promoting entry into fluid compartments, such as blood, saliva, glandular secretions, and lymph [16].

## WHY ARE EXOSOMES AN AREA OF INTEREST?

Given the wide range of molecular information carried forth from parent cells to secondary cells, exosomes may contribute to the characteristically aggressive metastatic profile of pancreatic cancer. Additionally, exosomes are secreted abundantly from pancreatic tumour cells. Exosomes also contain a plethora of information about tumour pathology and physiology [17]. Tumours can use this mode of communication to enhance the proliferative capacity, subtly changing the physiology of the host cell towards a pathological state [17]. Understanding the role of exosomes in pancreatic cancer may thus help address current gaps in the field, particularly regarding the detection and metastasic potential of early pancreatic lesions

## EXOSOMES AND METASTASIS

The metastasis of initial pancreatic lesions is preceded by the formation of a pre-metastatic ‘niche’, which is essential in facilitating the migration and proliferation of tumour cells [18]. Recent studies of the tumour micro-environment have found mounting evidence that exosomes may play a key role in the preparation of this micro-environment.

## EXOSOMAL PROTEINS IN METASTASIS

Exosomes primarily contain transport proteins and fusion proteins, as well as proteins for biogenesis of multivesicular bodies(MVBs) such as TSG101 [19]. Interestingly however, exosomal proteins are cell-type specific and are involved in cell-signaling pathways, two features which may indicate that exosomes are involved in the development of cancers [20]. Depending on the cell-of-origin, exosomal protein cargo may be oncogenic or act as tumour suppressors, thus affecting the tumour microenvironment.

Progression of pancreatic cancer is promoted by the heightened presence of pancreatic cancer initiating cells (PaCIC). These cells are marked by surface proteins such as CD44v6, CD44, MET, Tspan8, and CD133 [21, 22]. A study by Wang *et al*. demonstrated that these markers were incorporated and secreted within pancreatic cancer-derived exosomes [22]. Further studies have attempted to elucidate the functional importance of these proteins. [19]Using the rat pancreatic adenocarcinoma BSp73ASML line, Jung *et al*. [23] found that exosomes induce the settlement of the tumour cell line particularly in the lymph nodes and lungs. CD44v in exosomes was found to be a key mediator of this process, as it promotes the formation of a soluble matrix that facilitates the metastatic capacity of the tumour.

A key site of metastasis for PDAC is the liver, with fatal consequences [24]. Given the dire prognosis for PDAC, a heightened understanding of how metastasis occurs in crucial secondary sites is urgently required. A study by Costa-Silva *et al*. [25] found that exosomes also act as primers for metastasis in PDAC. The delivery of PDAC-derived exosomes to the livers of naïve mice increased the predisposition to metastasis. Kupffer cells in the livers of naïve mice took up the exosomes, leading to the creation of a fibrotic microenvironment. A notable feature of this fibrotic microenvironment that warrants further investigation is the elevated presence of macrophage migration inhibitory factor (MIF), a pro-inflammatory cytokine which may also promote tumour angiogenesis and proliferation [26]. Additionally, MIF may induce epithelial to mesenchymal transition (EMT) by which cells lose polarity and adhesion and become increasingly migratory [27]. Moreover, MIF was found to be highly expressed in cases of PDAC that later metastasized to the liver. It is hence suggested that the presence of MIF in exosomes may be a biomarker that can indicate the possibility of PDAC metastasis to the liver. According to a study by Yue *et al.* [28], the metastatic capacity of pancreatic lesions is also promoted by the presence of exosomes containing CD151 and tetraspanin 8, which recruit and activate integrins. The cells are thereby directed towards secondary tissues beyond the initial lesion.

A further characteristic of the premetastatic niche is the increased presence of myofibroblasts, which are heavily involved in the formation of collagen-rich scar tissue. Hence, fibrotic changes arise which alter the tissue architecture and extracellular matrix [29]. This has been associated with stromal alterations which enhance vascularisation, growth and metastatic capacity of solid cancers. A study of the effect of exosomes on target cell responses found that cancer-derived exosomes could in some cases trigger the conversion of fibroblasts to myofibroblasts, thus inducing pre-metastatic changes [30]. This was linked with increased expression of transforming growth factor beta (TGF-beta)on the surface of exosomes in association with betaglycan. Specifically, exosomes enhance the production of the fibroblast FGF2.

The regulation of the exosome secretion pathway has also been implicated in the progression of pancreatic cancer. A study by Wang *et al*. [31] studied RAB27A, a Rab GTPase integral to vesicle transportation, and tumour protein 53 (TP53), which is particularly involved in the secretion of exosomes. The study found that expression of RAB27A and TP53 was correlated with the clinical features of pancreatic cancer cases. The levels of both proteins were found to be significantly higher in cancerous sites, as opposed to benign tissues. Furthermore, increased RAB27A expression positively correlated with increased vascularisation and tumour progression. Increased RAB27A and TP53 in tandem were, overall, associated with poorer survival outcomes compared to controls with normal levels of protein expression. This leads to the idea that exosome secretion and trafficking from tumour cells may contribute to the formation of the tumour micro-environment. This further warrants the study of exosomes as a key biomarker for tumour progression and the differentiation of benign *versus* malignant cases. Thus, these findings highlight that exosomes are involved in the processes that prepare cellular environments for metastasis through a variety of means. It thus follows that if these preparatory molecules can be detected in exosomes from the tumour environment, this may serve as way to detect pancreatic lesions before they proceed to increasingly unmanageable states.

## EXOSOMAL MIRNA IN METASTASIS

miRNAs are non-coding RNAs of 19-25 nucleotides in length which regulate gene expression at the post-transcriptional level. This occurs through specific mRNA binding [32]. miRNAs have been reported to regulate key genes in oncogenesis and tissue differentiation. Additionally, the expression patterns of miRNA in various cells is highly tissue-specific [33]. Several recent studies have demonstrated that miRNA dysregulation is a feature of pancreatic cancer progression. Valadi *et al*. first described the presence of miRNA in exosomes [35]. It was later found that the transfer of miRNAs by exosomes had distinct biological effects in recipient cells [34]. In particular, the transfer of miRNAs by exosomes contributes to the formation of the premetastaic niche.

Using the BSp73ASML line, Rana *et al*. [18] further elucidated the role of exosomes in encouraging pre-metastatic niche formation by characterising the miRNA and mRNA profiles of exosomes. Supporting the results of *Jung et al.* [23] it was found that BSp73ASML-CD44-v7 knockdown cells had poor metastatic capacity in lymph nodes and lung tissues. Characterisation of exosomes uncovered that CD44v6 increased transcription and post-transcriptional modifications of particular genes and miRNA. miR-494 and miR-542-3p were found to be at higher levels in ASML(wt) exosomes, leading to increased matrix metalloprotease transcription and cdh17 downregulation. Cdh17 is a cadherin which leads to organisational changes within the GIT [18]. These miRNA increased the activity of various proteases, angiogenesis-promoting genes, and adhesion molecules among others, leading to an environment conducive to metastasis.

Similarly, Pang *et al*. [35] investigated the mechanism by which normal pancreatic fibroblasts can be converted to cancer-associated fibroblasts which lead to increased tumour invasiveness. It was found that microvesicles with elevated levels of miR-155 may contribute to increased fibroblast conversion. miR-155 targets and downregulates tumour protein p53-induced nuclear protein 1 (TP531NP1), conversely leading to the activation of the fibroblasts. It was thus concluded that the circulating miR-155 within microvesicles could lead to increased metastasis of pancreatic cancer.

A further way in which exosomes may prime secondary sites for metastasis may be by increasing inflammatory responses. A study by Fabbri *et al*. [36] found that miRNA secreted in exosomes from tumours go on to bind Toll-like receptors in immune cells. In turn, this leads to an inflammatory response that is conducive to the proliferation and metastasis of the original tumour. This further demonstrates that miRNA profiles are altered to create a tumour microenvironment favourable for invasion. As exosomes are a carrier for miRNA, this further illustrates the importance of exosomes as a potential biomarker for pancreatic cancer. It also illustrates that exosomes may be involved in preparing cellular micro-environments for tumour invasion.

## EXOSOMES AS METABOLIC REGULATORS OF CANCERS

Although the past decade has seen an explosion in research in the way tumours release exosomes to facilitate metastasis to secondary sites, there has been relatively little research on how exosomes modulate the metabolic status of the tumour itself. It has previously been shown that exosomes may reprogram the metabolic status of recipient cells. For example, exosomes may transfer GLUT transporters and enzymes involved in glycolysis [37]. Exosomes may also increase or decrease the level of oxidative phosphorylation or glycolysis occurring within the recipient cell by transfer of protein content, depending on the environment [38]. In the context of most cancers, fibroblasts are a prominent cells type at the forefront of metabolic regulation. Although classified as non-cancerous cells, fibroblasts are nonetheless crucial components of the tumour microenvironment. The activation of certain cancer-associated fibroblasts (CAFs) facilitates the growth and invasion of the tumour [39]. Notably, EVs and particularly exosomes derived from CAFs have been shown to promote the tumour micro-environment and enhance the metabolism of cancer cells to hence promote metastasis [41] [40]. Recently, Zhao *et al*. [40] demonstrated that CAF derived exosomes were a means of shuttling various metabolites to the cancer cells. This then led to a shift in cancer cell metabolism to favour cell growth and biosynthesis. Specifically, when prostate cancer cells were treated with CAF-derived exosomes there was a significant increase in glycolysis coupled with a reduction in oxidative phosphorylation. Culturing cells with exosomes also led to increased glucose uptake and secretion of lactate. These results were further reflected in the context of pancreatic cancer, in which pancreatic CAF-derived exosomes led to inhibition of mitochondrial function. Furthermore, the exosomal transfer of certain miRNA previously implicated in the modulation of oxidative phosphorylation (e.g. miR-22 and miR-25b) was shown to decrease oxygen consumption. In addition, exosomes from pancreatic CAFs contained whole metabolites, such as amino acids, palmitate, and lactate. These then contributed to the sustenance and growth of cancer cells under nutritionally-stressed conditions. Hence, exosomes were shown to transfer metabolites from the tumour microenvironment back to the cancer cells in a previously uncovered model of intracellular communication [40]. Further research into the molecular mechanisms by which exosomes are trafficked from the tumour micro-environment back to the cancer cells is warranted. In particular, it is necessary to study whether the crosstalk between CAFs and tumour cells by exosomes is organ-specific. Further understanding is also required regarding the role of CAF-derived exosomes in regulating particular processes in glycolysis.

Thus, these findings highlight that exosomes are involved in the processes that prepare cellular environments for metastasis through a variety of means. It follows that if these preparatory molecules can be detected in exosomes from the tumour environment, this may serve as way to detect pancreatic lesions before they proceed to increasingly unmanageable stages.

## EXOSOMES AS BIOMARKERS FOR PANCREATIC CANCER

Given the absence of non-invasive and accurate biomarkers in the clinic today, there has recently been significant interest in the use of exosomes as biomarkers for several cancers including pancreatic cancer. Not only is the use of exosomes as biomarkers non-invasive, exosomes are also stable and abundant. A study by Sarker *et al*. illustrated that the content of exosomes remains stable for several months, even after multiple freeze-thaw cycles [41]. The use of exosomes for screening large populations for early detection of pancreatic cancer is viable due to the stability of exosomes and availability of isolation methods, including differential ultracentrifugation [42]. Common methods of exosome isolation in the context of pancreatic-cancer derived exosomes are summarised in Table 1.

**Table 1:** Summary of exosome isolation methods and results of studies into the potential roles and uses of exosomes in pancreatic cancer

EXOSOME SOURCE	EXOSOME ISOLATION METHOD	RESULTS	REFERENCE
Primary cultures of murine pancreatic ductal adenocarcinomas (PKCY)	Ultracentrifugation	Enhanced TGF-beta signalling in Kupffer cells, correlated with poor patient outcomes. Accumulation of fibronectin leading to influx of bone marrow derived macrophages to the liver microenvironment. Upregulation of macrophage inhibitory factor.	[25]
Human pancreatic cancer cell line PANC-1 in culture	Ultracentrifugation	Exosomes taken up by dendritic cells. PC exosomes deliver miR-212-3p, leading to inhibition of RFXAP and MHC II expression and consequently contributing to immune tolerance. miR-212-3p may hence be crucial for PC progression.	[75]
Human pancreas cacrcinoma cell line Colo357	Successive centrifugation	Exosomes contain high levels of Hsp70/Bag-4; Hsp70 is involved in transmembrane protein transport, while Bag-4 binds to Hsp70 on its ATPase domain. Exosomes were enriched with Rab-4, indicating biogenesis and export via an intracellular route.	[76]
Human serum (PC, chronic pancreatitis, benign pancreatic tumour, non PC controls)	Sucrose-gradient centrifugation	PC exosomes were positive for PC initiating cell markers CD44v5, Tspan8, EpCAM, MET and CD104 This effect was not observed for non-malignant PC. miR-1246, miR-4644, miR-3976 and miR-4306 were expressed at higher concentrations in the majority of PC exosomes compared to controls.	[55]
Human serum (PC, benign pancreatic disease, and healthy donors) Human cell lines (e.g. HMLE, MIA Paca2, Panc-1)	Ultracentrifugation and ultrafiltration	Glypican-1 positive exosomes were better at identifying early pancreatic cancer compared to CA19-9 when distinct changes in pancreatic histology were absent.	[57]
Human serum (PC, healthy controls)	ExoChip (antigen based)	Significantly higher exosome capture in PC patients, compared to controls.	[73]
ASML (metastatic rat adenocarcinoma BSp73ASML) exosomes recovered in draining lymph nodes; cells cultured in serum-free medium	Centrifugation	ASML CD44vkd cells with poor metastatic potential were largely able to recover metastatic capacity when treated with ADMLwt or ASML-CD44vkd exosomes alongside ASMLwt conditioned medium. CD44v6 affects gene and miRNA transcription and content of exosomes. miR-494 and miR-542-3p were abundant in ASMLwt exosomes, which in turn increased matrix metalloprotease transcription..	[18]
Cell line supernatant (human pancreatic carcinoma or adenocarcinoma)	Ultracentrifugation and ultrafiltration	Exosomes decreased hairy and enhancer-of-split homolog-1 (Hes-1) expression, which is the target of Notch-1 signaling, and activated apoptosis. Exosomes inhibited cell proliferation by blocking key regulators of the Notch-1 pathway. Interactions occurred at lipid rafts.	[71]
PDAC cell lines	Ultracentrifugation and ExoQuick-TC purification	PDAC cells released exosmes in an integrin-b4 dependent manner. Integrin-b4 in exosomes, led to mislocalisation of plectin to the cell surface. Plectin was also found to enhance growth of the tumour in immunocompromised mice. Additionally, plectin was found to be key in the secretion of exosomes and contributed to the tumorigenic properties of exosomes.	[53]

As described above, exosomes contain a diverse molecular cargo comprising proteins and miRNA which is packaged into exosomes in a cell-specific manner. A review by Dillhoff *et al*. summarises the differential expression profiles of miRNA in various solid tumours [43]. Given their role in the development of cellular environments and the range of possible interactions with target genes, differential miRNA expression profiles hold potential as a biomarker for cancers and their progression. A study by Ali *et al.* sought to elucidate a differential miRNA profile for pancreatic cancer by comparing miRNA expression in pancreatic cancer patients, chronic pancreatitis patients, and healthy controls [44]. A range of miRNAs were found to be dysregulated in tumour samples. For example, miR-205, miR-155, miR-31 were upregulated in most tumour samples. Notably, upregulation of these miRNAs was inversely proportional to survival of patients. This supports the notion that miRNA profiles contribute to the oncogenesis of pancreatic cancer, and also that these profiles may be investigated further as a biomarker. Furthermore, a study by Gallo *et al*. [45] illustrated that compared to a supernatant that had been depleted of exosomes, exosome-rich samples had significantly higher miRNA content. Notably, some miRNAs were able to be detected in exosomes, but not in serum or supernatant. Consequently, if miRNA is to be used as a diagnostic biomarker for cancer, it is advantageous to study miRNA identity within exosomes. This is further highlighted in a study by Cheng *et al*. [46], who found that exosomes consistently provide a stable source of miRNA that can be used in diagnostic biomarker discovery. The exosomal source was significantly more enriched with miRNA compared to whole plasma and serum samples, thus further highlighting the potential benefits of using exosomes in particular as a biomarker. Additionally, the development of next-generation sequencing methods has greatly increased the feasibility of profiling and sequencing miRNA from exosomes from biological fluids. [46]

The idea that exosomes may be biologically active biomarkers of cancerous tissue was further supported in a study by Muller *et al*. [47] who studied new methods to recover exosomes free of contamination. It was found that exosomes from cancer tissue had aspects of increased biological activity, including elevated immune suppression through downregulated CD69 expression on CD4+ effector T cells. Beyond this, however, the study was important in illustrating that compared to non-cancerous controls, samples from cancer patients consistently yielded higher levels of exosomes. This illustrates that exosomes may be a key mediator of communication within and stemming from cancer tissue, and may be a potent biomarker for the pathobiology of the initial lesion.

The proteomic profile of exosomes derived from pancreatic lesions may be useful as a biomarker for diagnosing early cases of pancreatic cancer. A study by Klein-Scory *et al*. [48] illustrated that exosomes secreted from pancreatic cancer cells had a distinctive proteomic profile, allowing for possible use as a biomarker. In particular, this study found increased presence of membrane associated proteins, GTP-binding proteins, as well as glycoproteins. On the other hand, the exosomes lacked metabolic enzymes and proteins associated with proteasomes. Similarly, Adamczyk *et al.* [49] identified 3000 proteins secreted in exosomes derived from pancreatic cancer. A notable secretome was the epidermal growth factor receptor (EGFR), the binding of which is associated with increased activity of the carcinogenesis signal transduction pathway. Ligands of EGFR, including EGF and TGF-alpha, have been observed to be overexpressed in the majority of pancreatic cancer types [50]. When EGF binding to EGFR is enhanced, tumour aggressiveness apparently increases [51]. Through transduction mechanisms downstream of tyrosine kinase receptors, EGFRs lead to enhanced cell proliferation and migration and thus potentially metastasis. Secretomes were characterised using mass spectrometry and Western blotting. It was found that a 170 kDa EGFR along with a 65 kDa processed constituent (of C-terminal) is released in exosomes from the pancreatic cancer cells. A soluble 110 kDa soluble form is also released in secretomes through ectodomain shedding. These findings could potentially lead to the targeting of EGFRs in pancreatic cancer therapy. Additionally, the presence of specific EGFRs of given size may represent a novel biomarker in exosomes for pancreatic cancer. This was also supported in a study by Arscott *et al*. who found that EGFR isoforms are present at high levels in exosomes and thus may be used as a novel biomarker [52].

Additionally, it has been demonstrated that plectin, a scaffolding protein, can act as a biomarker for PDAC. A study by Shin *et al*. [53] found that plectin is found in exosomes secreted from the PDAC cells. Plectin is localised on the cell surface, anchored by integrin beta-4. It is thought that the pathogenesis of PDAC is promoted by the mislocalisation of plectin, leading to gain of function effects. In PDAC plectin is found on the cell surface, but in normal healthy conditions, plectin is located in the cytoplasm. Plectin has significant roles in the organisation of the cytoskeleton, particularly the linkage of intermediate filaments to transmembrane glycoproteins. The mislocalisation of plectin was found to lead to enhanced proliferation and metastatic potential of PDAC cells. The metastatic potentiation was largely attributed to the role of exosomes in transporting plectin around the cell. Moreover, plectin was also associated in a key role for the production of exosmes. Conversely, when plectin was knocked out in mouse models, the metastasis of the initial lesion was significantly reduced.

The ability of a distinctive proteomic profile to distinguish pancreatic cases *versus* non-cancerous controls was recently demonstrated in a cohort surveillance study by Potjer *et al* [54]. Serum samples were analysed for biomarkers in a cohort with the CDKN2A mutation, thus predisposed to cancer. A specific signature of proteins and peptides determined through mass spectrometry was used successfully to establish significantly higher discriminant scores in the cases, *versus* the controls. Furthermore, the success of this proteomic profile as a signature was compounded by the fact that detection of pancreatic cancer was not impeded by the presence of other cancers, such as melanoma.

More recently, Madhavan *et al*. [55] conducted a similar evaluation of the miRNA and proteomic profile of exosomes as a potential biomarker for pancreatic cancer. Earlier studies initially found that pancreatic cancer exosomes exhibit markers of cancer-initiating cells, including CD44v5, Tspan8, EpCAM, MET, and CD104 [22]. Building on this prior research, Madhavan *et al.* [55] conducted a blind study of serum from patients with pancreatic cancer, either enriched with exosomes or depleted of exosomes. Using flow cytometry, the exosomes were tested for the presence of the aforementioned cancer-initiating cell set. While serum exosomes from healthy controls or patients with benign disease failed to react, miRNA (miR-1246, miR-4644, miR-3976, miR-4306) and cancer initiating cell markers were at heightened levels in pancreatic cancer serum exosomes. Researchers found that when testing for these biomarkers in serum derived exosomes, sensitivity was greatly improved without compromising specificity. However, the earlier finding by Gallo *et al*. [45] that some miRNA may not be able to be effectively characterised in serum points to the need for future studies to assess the miRNA profile of exosomes in pancreatic cyst fluid.

The miRNA content of human serum exosomes derived from pancreatic cancer patients was also studied by Que *et al*. [56]. miR-17-5p was heightened in serum exosome samples from pancreatic adenocarcinoma patients. This elevation was positively correlated with the metastatic capacity and staging of the pancreatic cancer cases. Moreover, the advantage of using miRNA as a diagnostic marker compared to CA19-9 is highlighted by the ability of miRNA to distinguish between chronic pancreatitis and pancreatic cancer in this study. It was found that miR-21 was not only higher in pancreatic cancer derived exosomes compared to normal controls, it was also expressed at higher levels compared to chronic pancreatitis. This study highlights the potential for exosomal-miRNA to be used as a diagnostic biomarker for pancreatic cancer, particularly for its prognostic and aetiological advantages.

Exosome identification may at times be problematic due to difficulty in differentiating which exosomes are from the tissue itself, and which ones are from the tumour. However a study by Melo *et al*. [57] found that one way to identify cancer-cell derived exosomes may be through the presence of glypican-1 (GPC1). This is a glycoprotein which is present in enhanced quantities particularly on the cell surfaces of cancer-derived exosomes. The presence of GPC1 in exosomes were additionally used to distinguish normal control subjects from patients with benign pancreatic lesions. Furthermore, the glycoprotein was used to differentiate benign cases from early and late-stage cases. Despite the inability of magnetic resonance imaging to detect intraepithelial lesions in the pancreas, this study found that assessing circulating exosomes may provide a way to detect such lesions. Studying circulating exosomes also aided the identification of characteristic KRAS mutations, which are present in 70-95% of PDAC patients [58]. KRAS mutations involve a dysfunctional RAS protein, which means GTPase-activating proteins can no longer effectively convert the active GTP to the inactive GDP. This leads to constitutive activation of certain downstream pathways, including the PI3K and MEK/ERK pathways. In turn, this promotes cell proliferation, survival and differentiation [59]. A recent multicenter prospective study by Bournet *et al*. [58] using biopsies obtained from EUS-FNA found that in particular, the *KRAS* G12D mutant is promising as an independent marker and progress predictor of advanced pancreatic cancer cases.

While several studies have identified RNA and proteins in cancer-derived exosomes, a study by Kahlert *et al*. [60] assessed whether genomic DNA could be found in serum exosomes from patients with PDAC. The results of the study highlight the presence of fragments of double-stranded genomic DNA over 10 kb in size within the exosomes (pancreatic cell lines). The presence of genomic DNA is notable, as this can help identify the presence of mutations. For instance, this study also detected KRAS mutations as well as p53 mutations, thus aiding the characterisation of pancreatic cancer. Furthermore, genomic sequencing led to the conclusion that pancreatic cancer derived exosomes contain DNA across the full range of chromosomes. This reiterates the use of genomic DNA in exosomes as a way to develop the prognosis and management strategies for pancreatic cancer. Interestingly, it has been proposed that exosomes can be used as biological carriers for several molecules, including chemotherapy drugs as well specific proteins and RNAs.

## DRUG DELIVERY/TREATMENT FOR PANCREATIC CANCER USING EXOSOMES

Current treatments for pancreatic cancer have only been able to provide modest results in the past. While symptoms may be managed by some drugs, in most cases treatment of the underlying cause is not possible. Gemcitabine has been established as a standard of care [17]. However, the modes of therapeutic delivery have been expanded by the possibility of using the unique biological properties of exosomes to enhance treatment options.

Increasing evidence has accumulated for the potential uses of exosomes in cancer immunotherapy. Among this is increasing promise for exosomes as a method of ‘vaccination’, leading to attenuated tumour growth through enhanced immune responses. A study by Yang *et al*. [61] found that when IL-2 gene modifications (which have anti-tumour effects), are injected into mice with tumours using exosomes, inhibited tumour growth is observed. Researchers concluded that this was due to an antigen-specific Th1 polarised immune response mediated by cytotoxic T-lymphocytes. Enhanced anti-tumour responses using exosomes as a vehicle was also studied by Xie *et al*. [62] leading to a similar finding that immunisation using exosomes and cytokine genes induced significantly higher efficiency in the responses of T cells, particularly CD(+) T cells. Exosomes are also promising in their ability to act as vehicles for anticancer agents. A study by Aspe *et al*., [63] which involved induction of tetracyclin-regulated Survivin-T34A in exosomes, found that levels of apoptotic death were enhanced when these exosomes were plated onto pancreatic adenocarincoma cells. This effect was observed when Survivin-T34A was applied on its own, and also with gemcitabine. The ability of apoptosis-inducing agents to be incorporated in exosomes and delivered to tumour cells was also established in a study by Hosseini *et al*. [64]. Researchers constructed a novel structure which incorporated staphylococcal enterotoxin B onto the exosome. Cells derived from a pancreatic cell line were treated with varying concentrations of EXO/SEB and analysed using MTT assay and Hoechst staining. 0.5 and 2.5 mg/100 mL of EXO/SEB was sufficient to significantly enhance apoptosis in the recipient cells following a period of 24 hours.

Hence, the fact that exosomes capture a wide host of potentially biologically significant biomolecules indicates that they may be useful not only for understanding disease progression and as biomarkers, but also as potential carriers for treatments. This is particularly important for pancreatic cancer, in which current treatments cannot provide long-lasting benefits for large numbers of patients. Exosomes may hence provide a more targeted approach to disease management.

## CHALLENGES IN THE EXOSOME ISOLATION

Despite the various studies which have highlighted the potentially significant role of exosomes in both physiological and pathological conditions, the relevance of exosomes as clinical biomarkers or in disease intervention has stalled. This is largely due to challenges in isolating high concentrations of exosomes, especially without contamination from other EVs. One major challenge is the lack of a standardised method of exosome isolation and characterisation in the literature at present. This may potentially hinder advances in understanding the biological significance of exosomes in disease. Additionally, this significantly complicates the potential for exosomes to be used as routine clinical biomarkers or for other targeted uses [65]. It is therefore essential to establish a standardised technique for isolating high, relatively pure concentrations of exosomes.

Various techniques are currently used for exosome isolation, as seen in Table 1. The most common of these include ultrafiltration, density gradient separation, and ultracentrifugation [66]. Differential centrifugation is often a key component of isolation methods, designed to remove cell debris and other large vesicles to obtain a purer fraction of exosomes. This method has been applied to several body fluids, such as serum, urine and saliva, in addition to cell conditioned media [67, 68]. Sufficient rounds of centrifugation are especially important when considering body fluids, as various contaminants may be present. However, ultracentrifugation and centrifugation isolation methods may be problematic as they commonly provide a lower yield of exosomes compared to other methods, such as density gradient separation. This may be due to the formation of aggregates which hinder size separation mechanisms [69]. Additionally, centrifugation on its own may be inadequate due to contamination from other EVs, such as MVs. It has therefore been suggested that ultrafiltration may be a promising method for yielding purer populations of exosomes [70]. Ultrafiltration is a technique which purifies vesicles based on their size. This process commonly uses syringe filters. Exosomes are usually separated using a 0.22 um filter. Other ultrafiltration devices have emerged, also based on a filtration of vesicles so that vesicles with a maximum diameter of 0.22 um are retained. These include the Amicon ^®^ Ultra-15000 kDA tube [70].

Beyond centrifugation and filtration, density gradient separation has also been a common method of exosome isolation. This often involves the use of a sucrose gradient or cushion. However, although this method increases the purity of the exosomal fraction obtained, the yield may not be markedly higher than previously described methods. Another problem is that density gradient separation is highly time intensive [66]. Previous studies have often paired ultracentrifugation with ultrafiltration to improve the purity of the exosome population obtained while also minimising the time required for isolation [57, 71]. This may potentially be an effective way of isolating exosomes in an efficient manner, although strict standards must be applied to correctly categorise vesicles as exosomes.

Isolation devices have recently emerged as another way to sidestep the time intensive nature of high quality exosome isolation. For example, microfluidic devices separate and collect exosomes using micro-channels [72]. ExoChip, a particular microfluidic device, was recently described as a suitable method for recovering relatively pure concentrations of exosomes [73], as confirmed by Western blots and immuno-electron microscopy. Devices like ExoChip, if further confirmed to provide pure concentrations of exosomes, may also be high-throughput and relatively cost-effective ways of capturing exosomes [73].

There are hence an array of methods for isolating exosomes that are currently being used in the study of exosomes. It is necessary to consistently validate each of these methods according to stringent definitions of exosomes, for example those outlined by the International Society for Extracellular Vesicles [74]. Ideally, a standardised method for exosome isolation will be developed in the near future, thus maximising the relevance of laboratory-based studies of exosomes in the clinical setting.

## SUMMARY AND PERSPECTIVES

**Figure 1:**
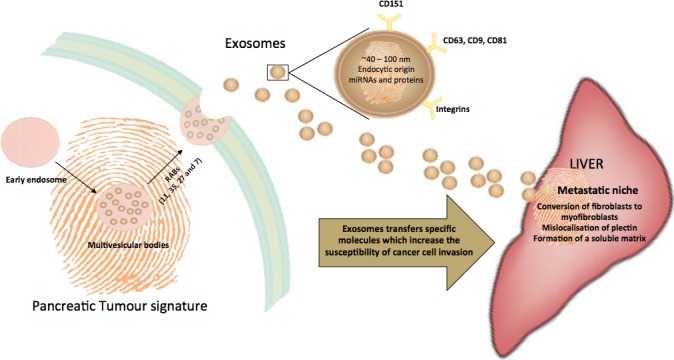
Trafficking of exosomes to the liver, a common site of metastasis for pancreatic cancers Exosomes are derived from the endocytic pathway and are released by exocytosis. Thus, exosomes encapsulate a unique, cell-specific ‘signature’ of the cellular environment. In cancer, exosomes facilitate the development of a metastatic niche by compromising the cellular matrix of the target tissue and making it more susceptible to invasion by tumour cells.

Exosomes are released by the tumour during cancer and their release may correlate with cancer outcome. *Via* a process of exosomal pancreatic-liver transfection, an array of receptors, proteins and/or oligonucleotides” that have been specifically pre-conditioned by the pancreatic tumour micro-environment may be delivered to target organs (e.g. liver). Exosomes hence prepare these sites for the cancer cell invasion (Figure 1). Given the current gap in the field for effective early diagnostic tools for pancreatic cancer, which thereby leads to low survival rates, it is crucial to harness the unique properties of exosomes that make them inextricably linked to metastasis. The content of exosomes is evidently largely shaped by the tumour cell as a means to transmit physiological information to shape a new pathological state. As such, exosomes can serve as a potential potential biomarker. Beyond increases in specificity and sensitivity through the use of exosomal biomarkers, the isolation of exosomes from patients is cheaper and less invasive than many present clinical diagnostics. It is now imperative to validate each of the identified biomarkers in large samples, as a lack of validation is a key barrier to clinical relevance. There is also a need to optimise and streamline high throughput exosome isolation methods to increase the efficiency of the process and hence work towards increased clinical relevance.
